# Fecal and serum metabolomic signatures and gut microbiota characteristics of allergic rhinitis mice model

**DOI:** 10.3389/fcimb.2023.1150043

**Published:** 2023-04-25

**Authors:** Zhen Chen, Shancai He, Yihan Wei, Yang Liu, Qingqing Xu, Xing Lin, Chenyu Chen, Wei Lin, Yingge Wang, Li Li, Yuanteng Xu

**Affiliations:** ^1^ Department of Otorhinolaryngology-Head and Neck Surgery, The First Affiliated Hospital of Fujian Medical University, Fuzhou, China; ^2^ Department of Otorhinolaryngology-Head and Neck Surgery, National Regional Medical Center, Binhai Campus of The First Affiliated Hospital of Fujian Medical University, Fuzhou, China; ^3^ Department of Otorhinolaryngology, Fuqing City Hospital Affiliated to Fujian Medical University, Fuzhou, China; ^4^ Department of Otorhinolaryngology, Fujian Children's Hospital, Fuzhou, China; ^5^ Allergy Center, The First Affiliated Hospital of Fujian Medical University, Fuzhou, China; ^6^ College of Life Sciences, Fujian Normal University, Fuzhou, China

**Keywords:** allergic rhinitis, gut microbiota, metabolomics, 16s rDNA sequencing, metabolite, α-linoleic acid, *Ruminococcus*

## Abstract

**Background:**

The etiology of allergic rhinitis (AR) is complicated. Traditional therapy of AR still has challenges, such as low long-term treatment compliance, unsatisfactory therapeutic outcomes, and a high financial burden. It is urgent to investigate the pathophysiology of allergic rhinitis from different perspectives and explore brand-new possible preventative or treatment initiatives.

**Objective:**

The aim is to apply a multi-group technique and correlation analysis to explore more about the pathogenesis of AR from the perspectives of gut microbiota, fecal metabolites, and serum metabolism.

**Methods:**

Thirty BALB/c mice were randomly divided into the AR and Con(control) groups. A standardized Ovalbumin (OVA)-induced AR mouse model was established by intraperitoneal OVA injection followed by nasal excitation. We detected the serum IL-4, IL-5, and IgE by enzyme-linked immunosorbent assay (ELISA), evaluated the histological characteristics of the nasal tissues by the hematoxylin and eosin (H&E) staining, and observed the nasal symptoms (rubs and sneezes) to evaluate the reliability of the AR mouse model. The colonic NF-κB protein was detected by Western Blot, and the colonic histological characteristics were observed by the H&E staining to evaluate inflammation of colon tissue. We analyzed the V3 and V4 regions of the 16S ribosomal DNA (rDNA) gene from the feces (colon contents) through 16S rDNA sequencing technology. Untargeted metabolomics was used to examine fecal and serum samples to find differential metabolites. Finally, through comparison and correlation analysis of differential gut microbiota, fecal metabolites, and serum metabolites, we further explore the overall impact of AR on gut microbiota, fecal metabolites, and host serum metabolism and its correlation.

**Results:**

In the AR group, the IL-4, IL-5, IgE, eosinophil infiltration, and the times of rubs and sneezes were significantly higher than those in the Con group, indicating the successful establishment of the AR model. No differences in diversity were detected between the AR and Con groups. However, there were modifications in the microbiota’s structure. At the phylum level, the proportion of Firmicutes and Proteobacteria in the AR group increased significantly, while the proportion of Bacteroides decreased significantly, and the ratio of Firmicutes/Bacteroides was higher. The key differential genera, such as *Ruminococcus*, were increased significantly in the AR group, while the other key differential genera, such as *Lactobacillus*, *Bacteroides*, and *Prevotella*, were significantly decreased in the Con group. Untargeted metabolomics analysis identified 28 upregulated and 4 downregulated differential metabolites in feces and 11 upregulated and 16 downregulated differential metabolites in serum under AR conditions. Interestingly, one of the significant difference metabolites, *α-*Linoleic acid (ALA), decreased consistently in feces and serum of AR. KEGG functional enrichment analysis and correlation analysis showed a close relationship between differential serum metabolites and fecal metabolites, and changes in fecal and serum metabolic patterns are associated with altered gut microbiota in AR. The NF-κB protein and inflammatory infiltration of the colon increased considerably in the AR group.

**Conclusion:**

Our study reveals that AR alters fecal and serum metabolomic signatures and gut microbiota characteristics, and there is a striking correlation between the three. The correlation analysis of the microbiome and metabolome provides a deeper understanding of AR’s pathogenesis, which may provide a theoretical basis for AR’s potential prevention and treatment strategies.

## 1 Introduction

Allergic rhinitis (AR) is a chronic inflammatory disease of nasal mucosa mainly mediated by immunoglobulin E (IgE), which happens in atopic individuals exposed to allergens (Greiner et al., 2011). AR seriously affects patients’ learning, work efficiency, sleep quality, and even varying mental disorders, causing huge social-economic losses (Bousquet et al., 2020). AR is a major chronic inflammatory disease of the respiratory tract, affecting 10%~20% of the world’s population (Brozek et al., 2010). Its incidence rate rises yearly in developed and rapidly developing countries (Wheatley and Togias, 2015). Although the pathogenesis of AR is not yet fully understood, it is often the result of immune dysregulation in response to various changes in environmental factors, including altered colonization by the gut microbiota.

More and more evidence reveals that patients with allergic diseases have experienced gut microbiota dysbiosis, including asthma (Chiu et al., 2019a; Johnson et al., 2020; Patrick et al., 2020), food allergy (FA) (Reddel et al., 2020; De Filippis et al., 2021), atopic dermatitis (AD) (Lee et al., 2022), and AR (Liu et al., 2020; Zhu et al., 2020; Su et al., 2021; Watts et al., 2021; Zhou et al., 2021), but the causal relationship between gut microbiota dysbiosis and the occurrence of allergic diseases is still unclear. The hygiene hypothesis suggests that reducing microbial exposure will aggravate allergic inflammation (Lambrecht and Hammad, 2017). Studies have also shown that altered gut microbiota composition, fecal metabolites, or serum metabolites in infancy may lead to allergic diseases in the later stage (Arrieta et al., 2015). However, changes in the host’s immune status may also affect the gut microbiota. Pang et al. (Pang et al., 2021) showed that allergic airway diseases (AAD) mice exhibited dysbiosis of the gut microbiota, accompanied by a decline in the variety and abundance of the Bacteroidetes phylum. Recent studies (Kim et al., 2019; Pang et al., 2021) have shown that AR condition may also lead to changes in gut microbiota composition in mice using 16S sequencing technology, indicating that AR status may also affect the gut microbiota.

However, gut microbiota analysis by 16S only explains the problem of “who is there”, and “what happened” needs further explanation from the fecal and serum metabolomics study. The multi-omics analysis of serum and feces has been widely used to explore the pathogenesis of allergic disease and even biomarkers (Irizar et al., 2021). Metabolomics can help fill the gaps where sequencing falls short by providing information on the metabolic interactions between the host, food, and gut microbiota (Marcobal et al., 2013). Some studies (Zhou et al., 2019; Shi et al., 2020) have demonstrated the altered serum metabolic signatures in AR patients and mice through serum metabonomic technology, including amino acid metabolism, L-tyrosine metabolism, and lipid metabolism. More research is being done on the pathophysiology of diseases using metabolomics and the microbiome together. Chiu et al.’s (Chiu et al., 2019a; Chiu et al., 2019b) analysis of children’s feces using NMR metabolomics and 16S rDNA sequencing technology revealed that children with AR had significantly lower fecal Dorea bacteria, Dialister bacteria, and histidine. However, the differential metabolites were not significantly related to the differential gut microbiota. In addition, Zhou et al. (Zhou et al., 2021) compared the differences between gut microbiota composition and SCFAs in the feces of AR patients and healthy people by 16S and metabolomics, indicating compositional and functional alterations of the gut microbiome in AR. Based on previous studies, AR has changes in the gut microbiota, intestinal microbial metabolites, and serum metabolites.

The population’s omics analysis can easily be affected by many factors such as diet, gender, and living habits, so we established a standardized animal model of AR and conducted multi-omics analysis on all of the mice’s feces and serum, combining 16S rDNA V3-V4 region sequencing for the investigation of the microbiome with LC-MS for the analysis of the fecal and serum metabonomic data. We analyzed how AR impacted the gut microbiota characteristics and metabolic patterns of feces and serum, after which we examined their relationships and involved pathways in more depth. Our study found that AR alters fecal and serum metabolomic signatures and gut microbiota characteristics, and changes in fecal and serum metabolic patterns are associated with altered gut microbiota in AR. Interestingly, we found that the intestinal and serum ALA of AR mice was significantly reduced, which may be related to the enrichment of *Ruminococcus* in the intestinal tract of AR mice. To our best knowledge, this is the first systematic analysis of the gut microbiota characteristics and metabolomic signatures of feces and serum in an AR mouse model. The alterations and correlations among the three will provide us with rich information about AR, gut microbiota, and host metabolism to explain how AR further affects mice’s fecal and serum metabolic signatures after affecting gut microbiota disturbance, which may provide a theoretical basis for the potential prevention and treatment strategies of AR.

## 2 Methods

### 2.1 Animals

Specific pathogen-free (SPF) female BALB/c mice (4 weeks of age) were purchased from Shanghai Shrek experimental animals Co., Ltd. The animals are raised under specific pathogen-free conditions in the experimental animal center of Fujian Medical University, maintained on a 12-h daylight cycle and with free access to commercial pelleted food and water *ad libitum*. All animal experiments were approved by the Institutional Animal Care and Use Committee (IACUC) of Fujian Medical University (IACUC number: FJMUIACUC2020-0106), and the animals’ care was provided following institutional guidelines.

### 2.2 Establishment of AR model in mice

**Figure 1:**
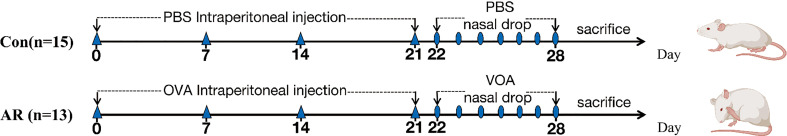
Protocol of the OVA-induced AR mice model.

According to previous literature, the OVA-alum AR model was established (Cho et al., 2019). Thirty BALB/c mice were randomly divided into the AR group (n=15) and the Con group (n=15). Briefly, each mouse in the AR group was sensitized by an intraperitoneal injection of 200 μl primary sensitizing liquid, including 40 μg of ovalbumin (OVA; grade V; Sigma, A5503), 100 μl of Imject™ Alum Adjuvant (40 mg/ml) (Thermo Fisher, 77161), and 100 μl PBS on days 0, 7, 14 and 21. In the AR group, each mouse’s nasal cavity was subjected to intranasal challenges with 10 μl of excitation liquid, including 100 μg of OVA and 10 μl of PBS, on seven consecutive days from 22-28. The Con group applied PBS instead of OVA on the same schedule. The protocol of the OVA-alum AR mice is shown in **Figure 1**.

### 2.3 Evaluation of nasal symptoms

After the last intranasal allergen challenge on day 28, the times of sneezes and rubs were counted for 15 minutes to evaluate allergic responses by blinded observers (Cho et al., 2019).

### 2.4 Sample collection

Samples of blood, colon contents, nasal mucosa, and colon mucosa were prepared 24 h after the last OVA nasal challenge. Blood was drawn from the ophthalmic artery, and serum was extracted by centrifugation at 4000 rpm (4°C for 10 minutes) and stored at -80°C. For the first time after mouse sacrifice, we cut off the colon specimen in the sterile operation, collected the feces (colon contents) in the sterile tube, and stored it at -80°C. The muscle tissue of the nose was removed, and the nasal cavity was fixed with 4% formaldehyde solution for 24 h at 37°C.

### 2.5 Detection of serum IL-4, IL-5, and IgE levels

Serum levels of IgE (Mskbio, 69-21184), IL-4 (Mskbio, 59-20069), and IL-5(Mskbio, 59-20070) in Con and AR groups were measured by ELISA kits, according to the manufacturer’s instructions. The absorbance was measured in a microplate reader at 450 nm. Triple pore detection was used to calculate the mean values.

### 2.6 Histopathological evaluation of nasal cavity and colonic tissue

The fixed nasal tissue was decalcified with a JYBL-I de-calcification solution (Solarbio, g2470). After 24 h, the nasal and colonic tissues were embedded in paraffin and cut into conventional sections at 4 μm. The sections (4-µm) were dewaxed, stained with hematoxylin (cat. no. 245880; Abcam) for 10 min, differentiated with 1% hydrochloric acid ethanol for 1 min, stained with eosin for 1 min, dehydrated with a series of ethanol concentrations (70, 80, 90 and 100%) ethanol for 10 sec, incubated with xylene for 1 min and sealed. In addition, other sections were dewaxed, soaked with 3% acetic acid for 3 min, stained with 1% Alcian blue (cat. no. 150680; Abcam) for 30 min, soaked with 3% acetic acid for 3 min, washed with water, oxidized with 0.5% periodate for 10 min, soaked in Schiff’s solution for 20 min and sealed.

### 2.7 Western blot of colonic tissue

The radioimmunoprecipitation assay (RIPA) buffer contains 25 mM Tris HCl (pH 7.2), 0.15M NaCl, 0.1% SDS, 1% Triton X-100, 1% sodium deoxycholate, and 1mM EDTA, was used to make tissue lysates. A bicinchoninic acid protein assay kit was applied to determine the protein content (Pierce). Proteins were transferred to a nitrocellulose membrane after being electrophoresed on sodium dodecyl sulfate-polyacrylamide gel. After blocking, a goat polyclonal antibody directed against a rabbit or mouse was used to identify total protein or phosphorylation. Using digital imaging equipment, protein bands were measured (UVtec).

### 2.8 LC-MS/MS analysis

Stool samples were diluted in water, ultrasonically mixed, and centrifuged at 14,000 rpm and 4°C for 20 min. The supernatant (0.5 ml), anhydrous sodium sulfate (0.3 g), 50% sulfate acid (10μl, and ether (1 ml) were added into a 4 mL Eppendorf tube and vortex-mixed. The mixture was centrifuged at 4,000 rpm and 4°C for 20 min. The serum samples (100 μl) were treated with methanol (200 μl), mixed, and centrifuged at 14,000 rpm and 4°C for 15 min. The supernatant was then analyzed by Ultra-High-Performance Liquid Chromatography-Tandem Time of Flight mass spectrometry (UPLC-Q-TOF/MS) to determine metabolites in the feces and serum.

### 2.9 16S rDNA high-throughput sequencing

16S rDNA high-throughput sequencing is an important method to study the composition and structure of the microbial community in the intestine by designing universal primers for PCR amplification using conserved regions and then sequencing analysis and strain identification of hypervariable regions. Total genome DNA was extracted from fecal samples using the CTAB/SDS method. 16S rDNA was amplified using a library of barcode-linked primers. The V3-4 hypervariable bacterial 16S rRNA gene region was amplified with the universal primer 338F (5’-ACTCCTACGGGAGGCAGCAG-3’) and 806R (5’-GGACTCANNGGGTA TCTAAT-3’). All PCR reactions were carried out in 30μl reactions with 15μl of Phusion^®^High-Fidelity PCR Master Mix (New England Biolabs), 0.2μM of forward and reverse primers, and about 10 ng template DNA. Thermal cycling consisted of initial denaturation at 98°C for 1 min, followed by 30 cycles of denaturation at 98°C for 10 s, annealing at 50°C for 30 s, and elongation at 72°C for 60 s. Finally, 72°C for 5 min. After PCR amplification, the mixture of PCR products was purified with AxyPrepDNA Gel Extraction Kit (AXYGEN). Following the manufacturer’s recommendations, sequencing libraries were generated using NEB Next^®^Ultra™DNA Library Prep Kit for Illumina (NEB, USA). Index codes were added, and the library was sequenced on an Illumina Miseq/HiSeq2500 platform, generating 250/300 bp paired-end reads. More detailed experimental steps and the analysis of the metagenomic data are described in supporting information. Sequence data associated with this project have been deposited in the NCBI Short Read Archive database (Accession Number: PRJNA948919).

### 2.10 Statistical analysis

The data are expressed as mean ± SD. The *t*-test and Wilcox’s test were used for comparison between groups. Correlation analysis was evaluated using a Spearman correlation analysis. *P* value < 0.05 was considered statistically significant. Analyses were performed using SPSS version 25.0, R(v3.6.0), and Python(v2.7).

## 3 Results

### 3.1 An OVA-alum AR model was successfully established

**Figure 2:**
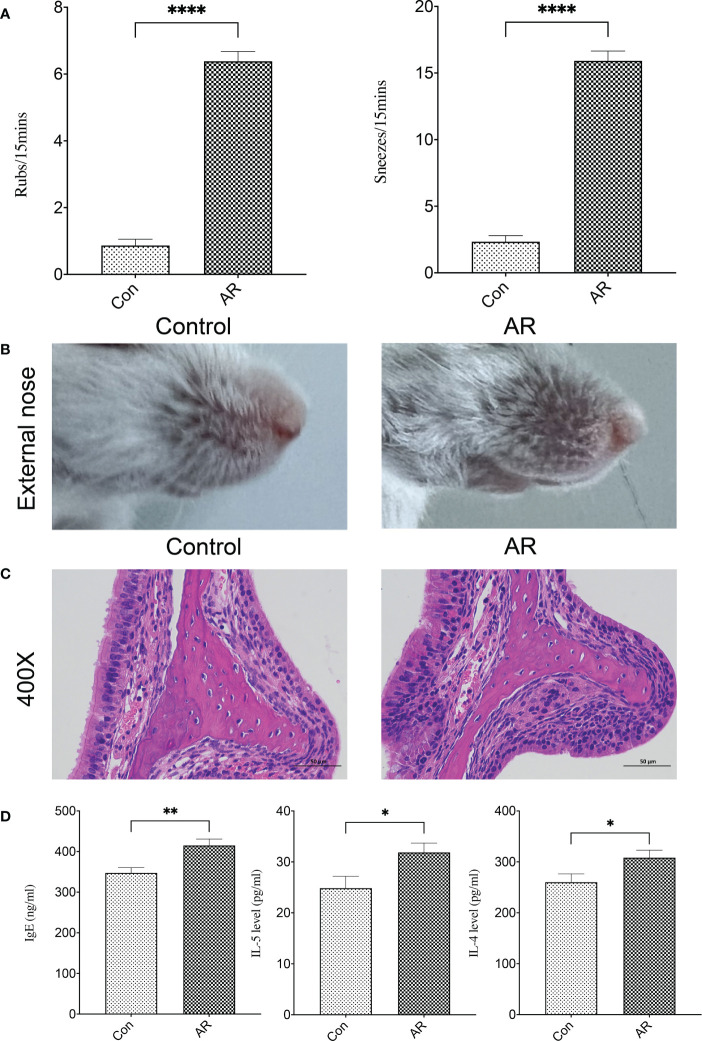
A reliable AR mouse model was established. **(A)** Nasal symptom; **(B)** Appearance of the external nose; **(C)** H&E staining of the nasal mucosa (400×); **(D)** IgE, IL-4, and IL-5 in serum. Con group(n=15) vs AR group(n=13). ns: *P >*0.05, *: *P*< 0.05, **: *P <*0.01, ****: *P <*0.0001.

Two mice in the AR group died of abdominal infection during the experiment, and the sneezes and rubs of the remaining 13 mice in the AR group were significantly higher than that in the Con group (**Figure 2A**). Compared with the Con group, AR mice showed obvious nose-scratching symptoms, with more sparse hair and redder skin around the nose (**Figure 2B**). H&E staining further showed that the nasal mucosa of AR mice experienced characteristic changes such as the disordered arrangement of epithelial cells, partial abscission, thickening of the basement membrane, and infiltration of the submucosal eosinophils (**Figure 2C**). The IgE, IL-4, and IL-5 in the serum of AR mice were significantly higher than that in Con mice (**Figure 2D**).

### 3.2 Altered gut microbiota composition in the OVA-alum AR model

**Figure 3:**
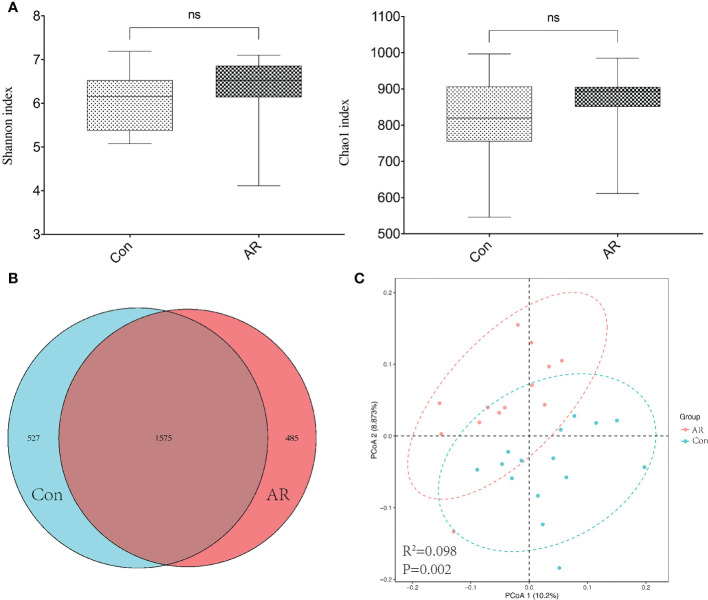
Diversity of gut microbiota in AR vs. Con groups. **(A)** The Chao1 and Shannon index; **(B)** Venn based on OTUs in AR vs. Con groups; **(C)** Principal coordinates analysis (PCoA). Dots of the same color represent each biological repetition in the group, and the distribution state of dots reflects the difference between and within the group. Con group(n=15) vs AR group(n=13). ns: *P >*0.05.

**Figure 4:**
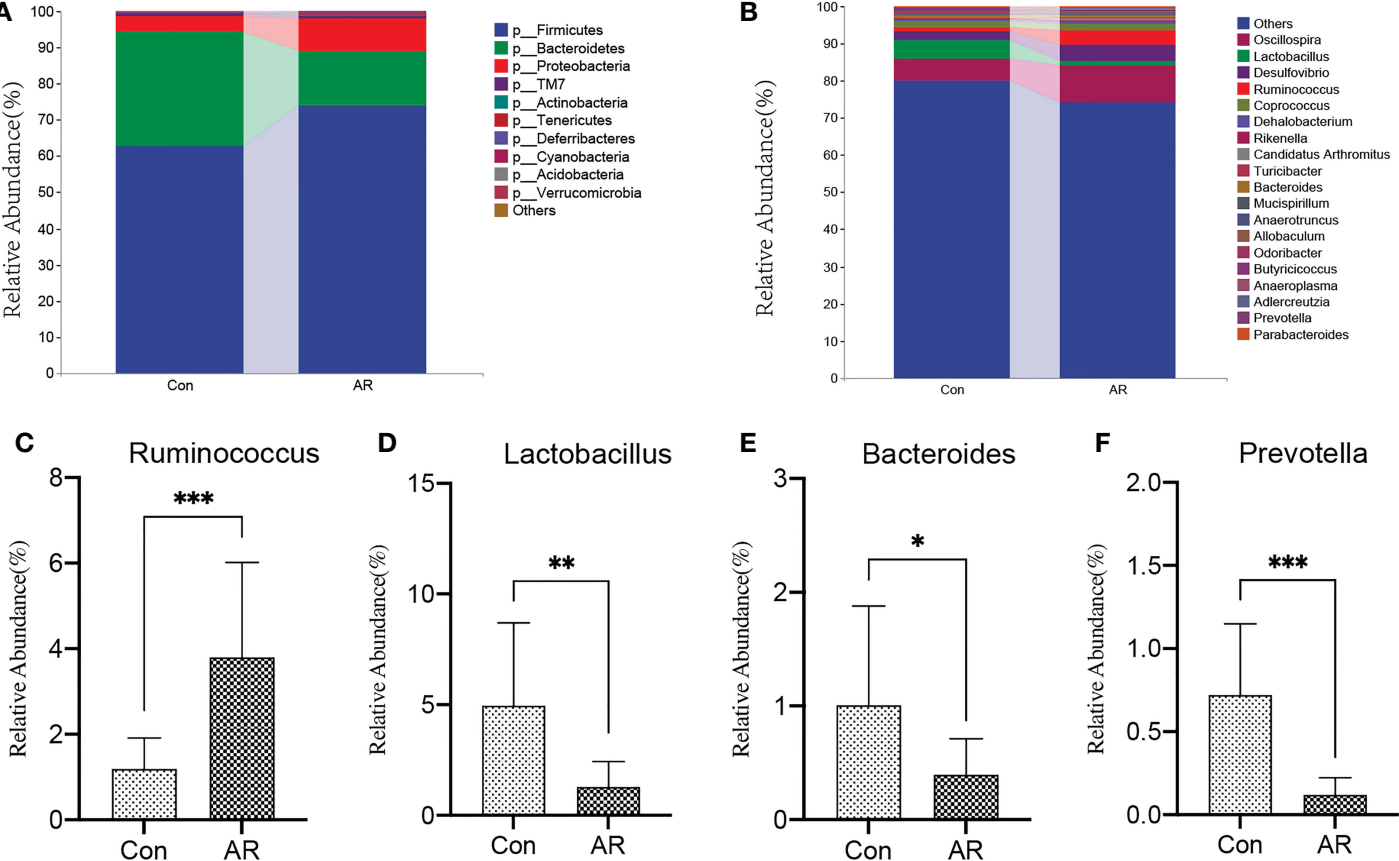
AR altered gut microbiota composition at phylum and genus levels. The top 10 phyla **(A)** and top 20 genera **(B)** of gut microbiota are colored differently, and the key genera, such as *Ruminococcus* **(C)**, *Lactobacillus* **(D)**, *Bacteroides* **(E)**, and *Prevotella* **(F)** are presented. Con group(n=15) vs AR group(n=13). *P <0.05, **P <0.01, ***P <0.001.

The sample size and amount of sequencing data available then were appropriate, as shown by the rarefaction curve (**Supplemental Figure 1**). Chao and Shannon’s indexes were important α-diversity indicators of gut microbiota. In the present study, we noted no differences in the Chao and Shannon indexes between the two groups (**Figure 3A**). By clustering the clean reads using a 97% criterion, a total of 2587 OTUs have been found (**Figure 3B**). Principal Coordinates Analysis (PCoA) showed significant differences in the microbial communities between the AR and controls (**Figure 3C**). In addition, the gut microbiota changed significantly at the phylum and genus levels. There was no significant difference between the Con and AR groups in Firmicutes (**Supplementary Figure 2A**). At the phylum level, compared with the Con group, the proportion of Proteobacteria in the AR model group was significantly higher, while Bacteroidetes were significantly lower (**Figure 4A**; **Supplementary Figures 2B, C**). The proportion of Firmicutes/Bacteroidetes in the phylum level of the AR group was higher than that of the Con group(**Supplementary Figure 2D**). **Figure 4B** shows that the top twenty gut microbiotas were enriched differently at the genus level in AR mice versus the Con group. *Ruminococcus* (**Figure 4C**) increased significantly in the AR group, while some genera, such as *Lactobacillus* (**Figure 4D**), *Bacteroides* (**Figure 4E**), and *Prevotella* (**Figure 4F**), decreased significantly in the AR group.

**Figure 5:**
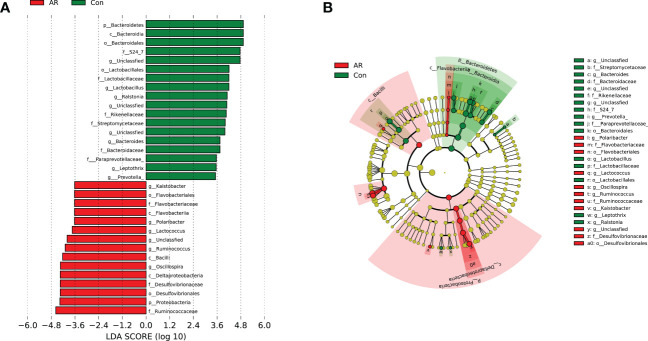
LDA scores map and cladogram (LDA fold=3.5 and *P <*0.05). The red node in the LDA value distribution histogram represents the microbial groups that play an important role in the AR group, and the green node represents the microbial groups that play an important role in the Con group. Only species with an LDA score > 3.5 are shown in the figure. **(A)** LDA score map. The histogram’s length represents the LDA value’s size; **(B)** Cladogram. The circles represent the phylum, class, order, family, and genus from the inside to the outside. Each small circle at different classification levels represents a classification at that level. The diameter of the small circle is proportional to the relative abundance.

Linear discriminant analysis Effect Size (LEfSe) analysis is a linear discriminant analysis (LDA) of samples according to different grouping conditions and taxonomic compositions to determine the communities or species that express significant differences in sample division (Segata et al., 2011). LEfSe analysis was used to explore the gut microbiota with significant statistical significance and biological correlation between the AR and Con group, which is represented by the LDA score map (**Figure 5A**) and branching diagram (**Figure 5B**) with LDA fold = 3.5 and *P <*0.05. Several microbiotas, such as p_Proteobacteria, and g_*Ruminococcus*, increased significantly in the AR group. Several microbiotas, such as p_Bacteroidetes, g_*Lactobacillus*, g_*Bacteroide*s, and g_*Prevotella*, were enriched significantly in the Con group.

### 3.3 Altered fecal and serum metabolomic signatures in the OVA-alum AR model

**Figure 6:**
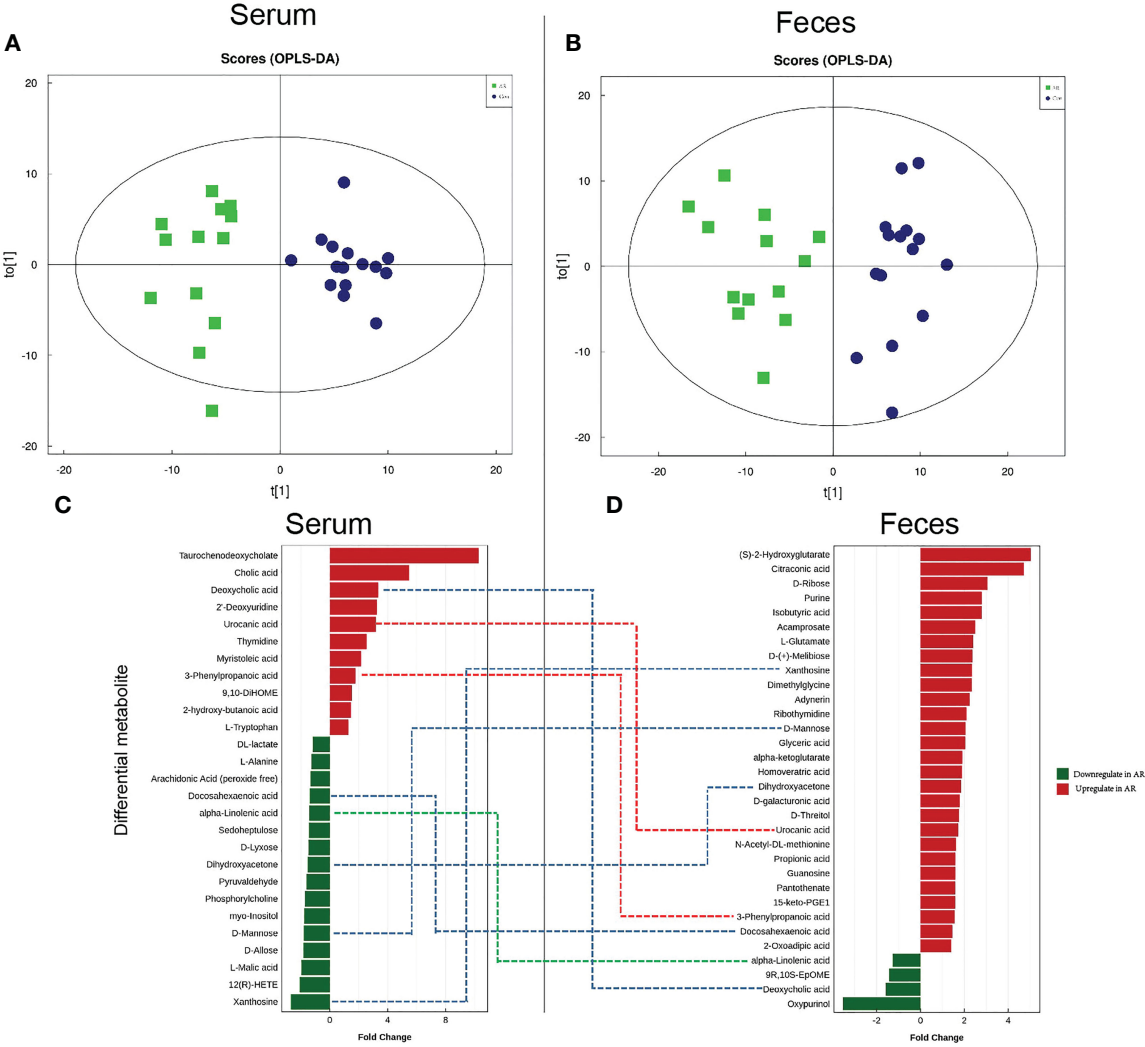
Characteristics of metabolomics in serum and feces. OPLS-DA score plots in serum metabolomics **(A)** and fecal metabolomics **(B)**. Dots of the same color represent each biological repetition in the group, and the distribution state of dots reflects the difference between and within the group; Histogram of significant differential serum metabolites **(C)** and fecal metabolites **(D)**. The red histogram shows the metabolites that are significantly elevated in the AR group (Fold change >1). The green histogram shows the significantly reduced metabolites in the AR group (fold change>1). In the middle of **Figure 6C** and **Figure 6D**, the red dotted line represents the metabolites increased in feces and serum, the green dotted line represents the metabolites decreased in feces and serum, and the blue dotted line represents the metabolites increased or decreased inconsistently in feces and serum.

Considering the distinct composition of gut microbiota between the AR and Con groups, we further performed metabolomics of fecal and serum samples from mice using UPLC-Q-TOF/MS in negative ion mode (ES-). The serum and feces metabolomic signatures were remarkably separated from the Con group in Orthogonal Partial Least Squares Discriminant Analysis (OPLS-DA) score plots (**Figures 6A, B**). OPLS-DA is an analysis method to correct partial least squares discrimination analysis, which can filter out noise unrelated to classification information and improve the analytical ability and effectiveness of the model. The validation of this model showed no overfitting phenomenon, indicating that the model could describe the samples well and be applied in further data analysis. Subsequently, the differential metabolites with VIP>1 and *P* value <0.05 in OPLS-DA analysis were further analyzed.

AR disrupts the serum metabolome. We ultimately identified 27 differential metabolites, with the changing trend illustrated by the histogram (**Figure 6C**). Compared with the Con group, 16 serum differential metabolites, such as DL-lactate, L-Alanine, *α*-Linolenic acid (ALA), and D-Mannose, were significantly reduced in the AR group. In contrast, 11 serum differential metabolites, such as Taurochenodeoxycholate (TCDCA), Cholic acid, Deoxycholic acid, Urocanic acid (UCA), 3-Phenylpropanoic acid, and L-Tryptophan, were increased significantly in the AR group. We listed the Log2FC, *P*-value, and VIP values of the serum differential metabolites in **Supplemental Table 1**. Compared with Con group, the levels of Oxypurinol, Deoxycholic acid, 9R,10S-EpOME, and alpha-Linolenic acid were decreased and 2-Oxoadipic acid, 3-Phenylpropanoic acid, etc were increased.

AR disrupts the fecal metabolome. We identified 32 differential metabolites, with the alterations illustrated by the histogram (**Figure 6D**). Compared with the Con group, 4 fecal differential metabolites, such as ALA, and Deoxycholic acid, decreased significantly in the AR group; however, 28 fecal differential metabolites, such as Purine, Isobutyric acid, D-Mannose, and Propionic acid, were increased significantly in the AR group. We listed the Log2FC, *P*-value, and VIP values of the serum differential metabolites in **Supplemental Table 2**. Compared with the Con group, the levels of Xanthosine, 12(R)-HETE, L-Malic acid, etc, were decreased and L-Tryptophan, 3-Phenylpropanoic acid, etc, were increased.

### 3.4 Correlation among key differential genera, differential fecal metabolites, and differential serum metabolites

**Figure 7:**
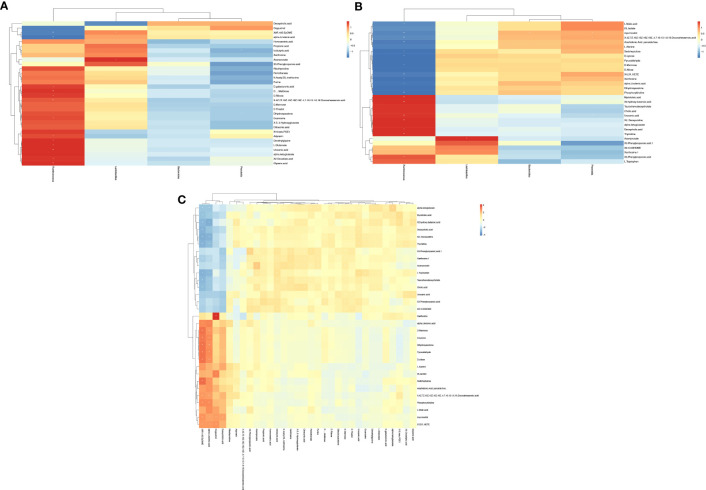
Effects of AR on the gut microbe, fecal, and serum metabolism. The predictive functional pathway (level 3) of gut microbes **(A)** and the enrichment of serum metabolic pathways **(B)** and fecal metabolic pathways **(C)** in AR vs. Con.

We analyzed correlations among key gut microbiota, differential fecal metabolites, and differential serum metabolites based on Spearman analysis and constructed heat maps. The relationship between differential fecal metabolites and differential genera is shown in **Figure 7A**. We found that the AR-enriched genus *Ruminococcus* is significantly negatively related to differential fecal metabolites, such as ALA, while that is significantly positively related to differential fecal metabolites, such as Glyceric acid and L-Glutamate. The correlation between differential serum metabolites and key differential genera is shown in **Figure 7B**. The AR-enriched genus *Ruminococcus* is considerably adversely associated with differential fecal metabolites, such as ALA, Phosphorylcholine, Glyceric acid, and L-Glutamate, which are strongly positively related to *Ruminococcus*. **Figure 7C** depicts the connection between distinct fecal metabolites and differential serum metabolites. We noticed that many differential fecal metabolites are positively related to differential serum metabolites.

### 3.5 Effect of AR on functional pathways

**Figure 8:**
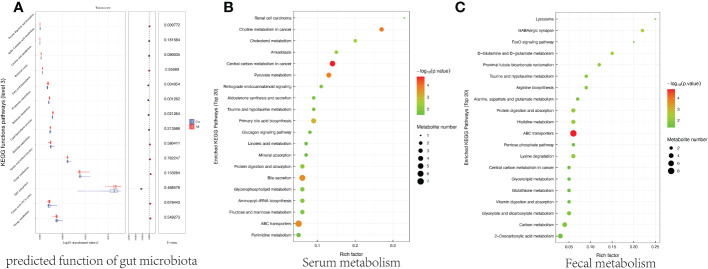
Correlation among key differential gut microbiota (genus level), differential fecal metabolites, and differential serum metabolites. **(A)** Correlation between key differential gut microbiota (genus level) and differential fecal metabolites. **(B)** Correlation between key differential gut microbiota (genus level) and differential serum metabolites. **(C)** Correlation between differential serum metabolites and differential fecal metabolites.

PICRUSt2 (Phylogenetic Investigation of Communities by Reconstruction of Unobserved States) is a tool for predicting bacterial community function based on species (Douglas et al., 2020), and we used it to predict gut microbiota function. **Figure 8A** showed the two groups’ significantly different Kyoto Encyclopedia of Genes and Genomes (KEGG) functional pathways and focused on the differences in metabolic functions. Several metabolic pathways in the AR mice model were significantly enriched, including Lipid biosynthesis proteins, Propanoate metabolism. To further analyze the effects of AR on metabolism, metabolites from both groups, the top 20 serum metabolic pathways, and the top 20 fecal metabolic pathways are shown in **Figures 8B**, **C**, respectively. **Figure 8B** showed several metabolic pathways in the AR group were highly enriched, including ABC transporters, Bile secretion, and central carbon metabolism. **Figure 8C** also demonstrated that ABC transporters were highly enriched in the AR group, while several metabolic pathways, including Carbon metabolism and Lysine degradation, were significantly enriched in the Con group.

### 3.6 Inflammatory infiltration and NF-κB protein of the colon increased in AR mice

**Figure 9:**
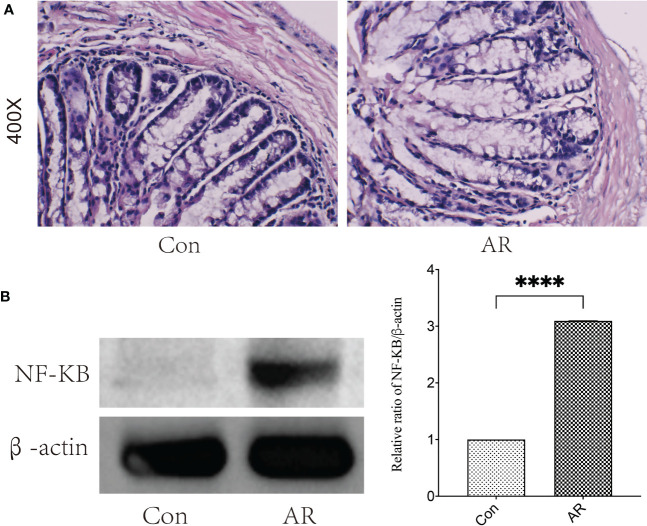
The severity of injury and inflammation in colon tissue increased in AR mice. **(A)** H&E staining of colonic mucosa (400×) **(B)** The protein expression of NF-κB was detected by western blotting. ****P < 0.0001.

A recent study (Bai et al., 2022) revealed that the inflammation of colonic tissue in AR mice increased, and the gut microbiota alerted, indicating that inflammation of intestinal mucosa may affect gut microbiota and its metabolites, so we observed the content of colonic NF-κB protein by western blot and the colonic histological characteristics by H&E staining. Our study showed that inflammatory infiltration (**Figure 9A**) and NF-κB protein (**Figure 9B**) of the colon increased considerably in the AR group compared to the Con group.

## Discussion

Based on the current discussion on the pathogenesis of AR, clinical treatment for AR includes environmental control, drug treatment, immunotherapy, and surgical intervention (Wheatley and Togias, 2015). However, there still are problems, such as low long-term treatment compliance, unsatisfactory therapeutic outcomes, and a high financial burden on patients. Therefore, exploring new mechanisms and brand-new possible preventative or treatment methods is necessary from different perspectives. In this study, we established a standardized AR mice model and conducted multi-omics analysis on the mice’s feces and serum. To our best knowledge, this is the first systematic analysis of the gut microbiota characteristics and metabolomic signatures of feces and serum in an AR mouse model. Our study found that AR disrupts gut microbiota characteristics, feces metabolomic signatures, and serum metabolomic signatures. Our findings especially outlined the relations of differential gut microbiota, fecal metabolites, and serum metabolites in AR. These studies supported that allergic rhinitis is a systemic allergic disease, which may alter the gut microbiota, fecal metabolism, and serum metabolism, and there is a certain correlation between the three.

Our study showed that AR alters gut microbiota composition. The proportion of the phyla Firmicutes in the AR group was higher, while the phyla Bacteroidetes significantly decreased in AR, consistent with a recent study about AR adult patients and mice (Pang et al., 2021). Many Bacteroides are symbiotic species highly adapted to the gastrointestinal tract, with very broad metabolic potential, and can be selectively recognized by the host immune system through specific interactions (Rajilić-Stojanović and De Vos, 2014). Recent studies (Pang et al., 2021) showed that feeding with Bacteroides (B.) the taiotaomicron alleviated the symptoms of OVA-induced OVA-induced airway hyperresponsiveness in mice. The ratio of Firmicutes/Bacteroidetes and Proteobacteria increased in the AR group. The increased ratio of Firmicutes/Bacteroidetes has been considered a sign of gut microbiota dysbiosis (Chen et al., 2022), also found in patients with irritable bowel syndrome (Hou et al., 2022). In addition, this study also showed that the proportion of Proteobacteria in the phylum level increased significantly in AR, which was also found in the other AR’s gut microbiota study (Kim et al., 2019). Most Proteobacteria are gram-negative bacteria, and their outer membrane contains many lipopolysaccharides (LPS) molecules, one of the most effective inflammatory inducers (Agus et al., 2016). In addition, our previous study also showed that Proteobacteria was associated with the aggravation of allergic rhinitis (Chen et al., 2022).

Our study showed that AR mice have a higher relative abundance of *Ruminococcus*, which is comparable to the results of previous studies (Chua et al., 2018; Chen et al., 2022). In addition, Chua et al. (Chua et al., 2018) found that the airway inflammation of mice was promoted by feeding *Ruminococcu* to mice, which was characterized by the expansion of T-helper 2 cells in the colon and lung and infiltration of eosinophils and mast cells. *Ruminococcus* is considered to be associated with childhood respiratory diseases (Alcazar et al., 2022) and be one of the key genera that aggravate allergic diseases. Previous studies reveal that decreased relative abundances define the gut microbiota dysbiosis of allergic diseases. Our research also showed that the genera *Lactobacillus* decreased significantly in the AR group. *Lactobacillus* is the main member of the lactic acid bacteria group, which can convert sugars to lactic acid.

Interestingly, we found that serum DL-lactate decreased significantly through serum metabolomics. When AR attacks, the body’s energy consumption increases, decreasing lactate, an intermediate product of glucose metabolism *in vivo* (Adeva-Andany et al., 2014). The depletion of *Lactobacillus* and serum DL-lactate may be a feature of the AR condition. Studies have supported that *Lactobacillus* or its metabolic products lactate supplementation could relieve allergic diseases (Kalliomäki et al., 2001; Selle and Klaenhammer, 2013; Kozakova et al., 2016; Rezazadeh et al., 2018; Esber et al., 2020; Suzuki et al., 2020) by improving the intestinal epithelial barrier, reducing allergen-specific IgE, and increasing regulatory cytokine TGF-β. The present study provided evidence for the value of *Lactobacillus* in the potential treatment of AR.

Metabolomics, which can explore the overall changes of many metabolites in a given sample by analyzing the endogenous or exogenous small molecular metabolites of organisms, has become a powerful tool for studying the etiology of allergic diseases and identifying potential biomarkers (Irizar et al., 2021). Our untargeted metabolomics analysis identified 28 upregulated and 4 downregulated differential metabolites in feces and 11 upregulated and 16 downregulated differential metabolites in serum under AR conditions. We found that L-alanine was significantly reduced in the serum of AR mice, consistent with the population study (Zhou et al., 2019). L-alanine is a non-essential amino acid that can facilitate the metabolism of sugar and acid and provide energy for the body. Alanine levels decreased significantly during the seizure stage, which may also reflect the upregulation of glycolysis. Our study supported the increase of energy metabolism in AR mice, and L-alanine may be one of the key metabolic links.

Importantly, we found that ALA decreased consistently in the feces and serum in AR mice. ALA is the main Omega-3 polyunsaturated fatty acid (Ω-3 PUFA), the essential fatty acid that humans should obtain from their diet. More and more evidence shows that ALA can inhibit inflammation and be beneficial in various inflammation-related diseases, such as inflammatory bowel disease, rheumatoid arthritis, and asthma (Liu et al., 2021). Under desaturase and extender, ALA can be transformed into DHA, which competes with the synthesis of inflammatory Arachidonic Acid, resulting in decreased prostaglandin E synthesis and inhibiting the production of cytokines and IgE related to allergy (Miyake et al., 2007). Population cohort studies (Mihály et al., 2014; Magnusson et al., 2018) have also shown that serum ALA in patients with allergic diseases is decreased, and supplementation of ALA may ameliorate bronchial asthma features in ovalbumin-sensitized rats (Boskabady et al., 2019). In addition, a recent study showed that ALA screened by molecular docking attenuates inflammation by regulating Th1/Th2 imbalance in OVA-induced AR mice (Ren et al., 2022). A previous study revealed that Ω-3 PUFAs (including ALA) maintain intestinal health by reducing oxidative stress and NF-κB mediated inflammation in immune cells and intestinal cells (Gutiérrez et al., 2019). Our study also showed that the NF-κB protein and inflammatory infiltration of the colon increased considerably in the AR group. That evidence indicated that gut microbiota regulates the intestinal and host immune system through intestinal-related metabolites, such as ALA (Fu et al., 2021).

Correlation analysis was carried out to comprehend better how metabolites and gut microbiota interact. Key differential genera, such as *Ruminococcus*, have multiple interactions with key differential fecal metabolites. Our study found that *Ruminococcus* negatively correlated with ALA and Arachidonic acid. Interestingly, ALA in serum and feces decreased consistently. More and more evidence shows a correlation between ALA and gut microbiota (Fu et al., 2021). ALA can affect gut microbiota; In turn, gut microbiota will also affect the metabolism and absorption of ALA. We supposed that OVA-induced AR might lead to gut microbiota dysbiosis (e.g., increased *Ruminococcus*), resulting in decreased ALA in the gut and serum. In return, the decreased fecal and serum ALA may also aggravate the host immune imbalance, which may be due to the change of intestinal mucosal immune state under the allergic inflammatory state of the host (Bai et al., 2022), thus affecting the composition of gut microbiota. However, it still needs to be confirmed by gut microbiota transplantation or flora-feeding experiment.

Even though standardized mouse experiments have greatly reduced the effects of individual heterogeneity, lifestyle, and diet, the different experimental schemes, mouse strains, and sampling locations remain challenging. In addition, the identified associations’ details remain to be interpreted and validated due to limited knowledge from relevant studies to date. Ideally, corroboration of these microbe-metabolite interactions and their role in AR pathogenesis will be achieved by further *in vivo* and *in vitro* experiments.

## Conclusion

**Figure 10:**
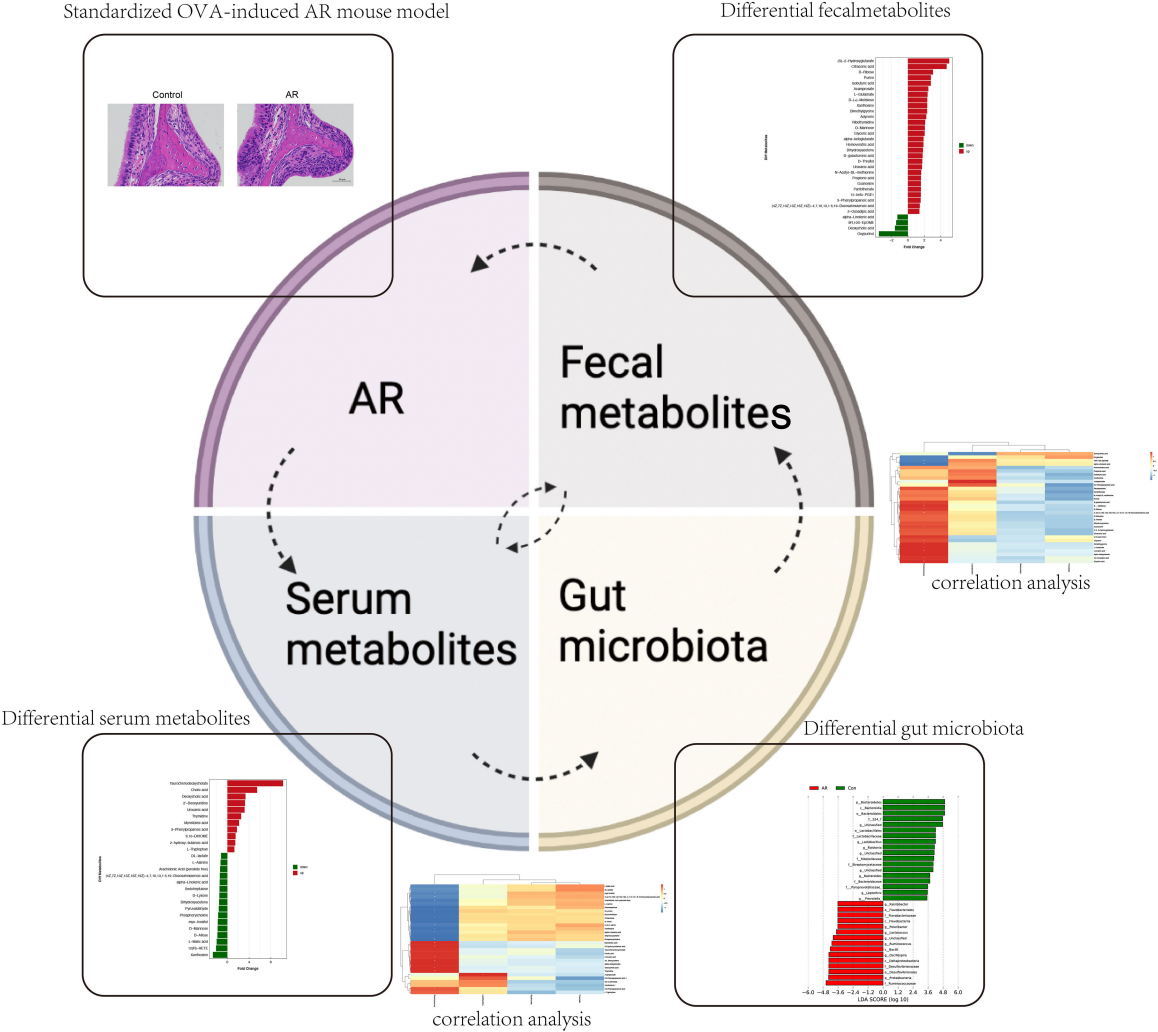
The OVA-alum AR model disturbs gut microbiota composition and alters the fecal and serum metabolic patterns and the correction among the three.

In summary, integrating these multi-omics findings, we characterized the landscapes of altered gut microbiota and metabolites in fecal and serum in AR mice and showed how these disturbed signatures were involved in the onset of AR (**Figure 10**). Metabolic gut microbiota products might link the gut and systemic immune homeostasis in AR. Our findings would advance our understanding of the pathophysiology of allergic rhinitis from different perspectives and explore brand-new possible preventative or treatment initiatives.

## Data availability statement

The datasets presented in this study can be found in online repositories. The names of the repository/repositories and accession number(s) can be found below: https://www.ncbi.nlm.nih.gov/,PRJNA948919.

## Ethics statement

The animal study was reviewed and approved by the Institutional Animal Care and Use Committee (IACUC) of Fujian Medical University.

## Author contributions

ZC and SH designed the project, performed the experiment, drafted the manuscript, and they contributed equally. YL, YWe, CC, and QX performed the experiment and collected the study data. YL and YWa analyzed the data. LL and YX conceived the study, got administrative support, and they contributed equally. All authors contributed to the article and approved the submitted version. 
