# Supplementary material for: Fecal and serum metabolomic signatures and gut microbiota characteristics of allergic rhinitis mice model
Source: Front Cell Infect Microbiol. 2023 Apr 25;13:1150043. doi: 10.3389/fcimb.2023.1150043 (PMC10167002; doi:10.3389/fcimb.2023.1150043)
Supplement: Supplementary file 1 [file DataSheet_1.docx]

Supplementary Material

# Supplementary Figures and Tables

## Supplementary Figures


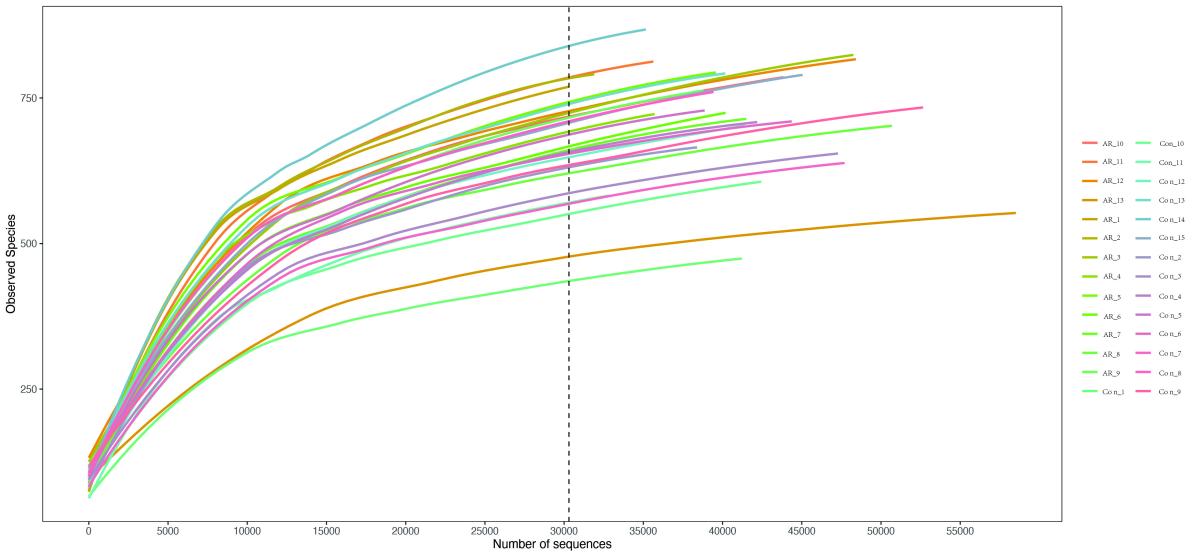


**Supplementary Figure 1.** Rarefaction curve based on OUT count in Con and AR groups.


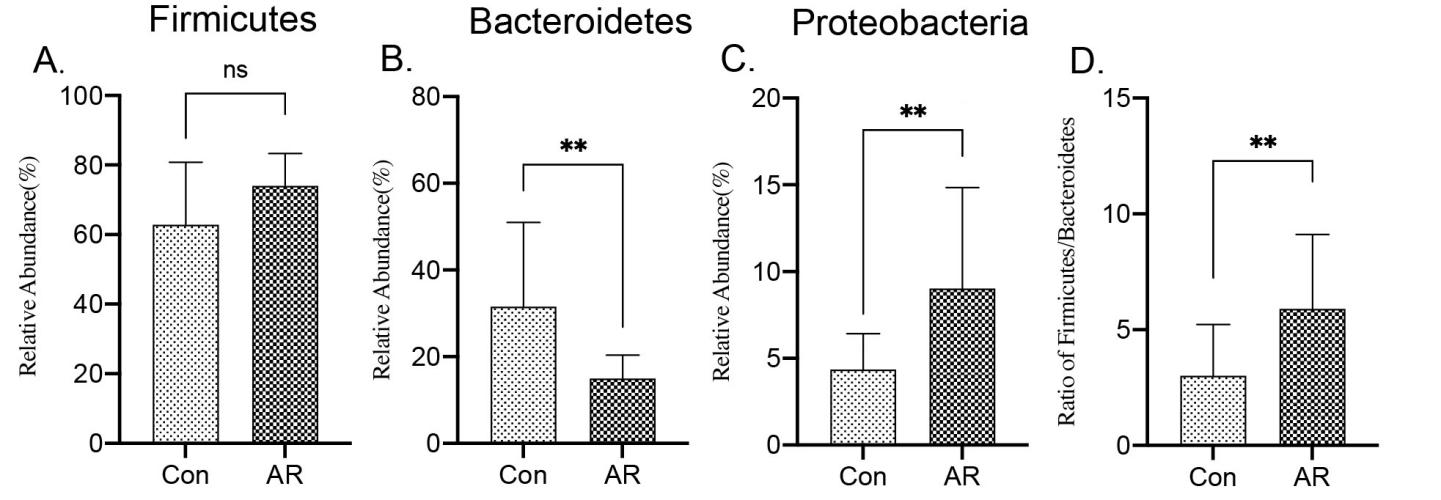


**Supplementary Figure 2. The difference of phyla level between Con and AR groups.** A. Firmicutes, B.Bacteroidetes, C.Proteobacteria, D.Ratio of Firmicutes/Bacteroidetes. ns: *P*>0.05, **: *P*<0.01.

## Supplementary Tables

**Supplementary Tables 1**|Differential fecal metabolites.

| **Name** | **VIP** | **Fold change** | ***P* value** | **Up Down** |
| --- | --- | --- | --- | --- |
| **Oxypurinol** | 4.199762456 | 0.283362467 | 0.022175574 | Down |
| **Deoxycholic acid** | 3.012273872 | 0.632309344 | 0.035620886 | Down |
| **9R,10S-EpOME** | 7.755273836 | 0.701052756 | 0.019646186 | Down |
| **alpha-Linolenic acid** | 6.562307461 | 0.781729232 | 0.028269424 | Down |
| **2-Oxoadipic acid** | 9.206371902 | 1.404634844 | 0.003473406 | Up |
| **(4Z,7Z,10Z,13Z,16Z,19Z)-4,7,10,13,1 6,19-Docosahexaenoic acid** | 7.513285655 | 1.451906344 | 0.034406183 | Up |
| **3-Phenylpropanoic acid** | 3.749171291 | 1.554478384 | 0.043526583 | Up |
| **15-keto-PGE1** | 1.376602749 | 1.580608718 | 0.007394559 | Up |
| **Pantothenate** | 3.967975349 | 1.58354287 | 0.012698089 | Up |
| **Guanosine** | 1.576695955 | 1.587780451 | 0.022851276 | Up |
| **Propionic acid** | 3.327497413 | 1.591881289 | 0.006380592 | Up |
| **N-Acetyl-DL-methionine** | 2.017492957 | 1.61605541 | 0.013367826 | Up |
| **Urocanic acid** | 1.834881927 | 1.719507729 | 0.000645282 | Up |
| **D-Threitol** | 1.322598364 | 1.75285373 | 0.000736756 | Up |
| **D-galacturonic acid** | 1.190135528 | 1.792095237 | 0.013924696 | Up |
| **Dihydroxyacetone** | 1.30121876 | 1.840083767 | 0.000261786 | Up |
| **Homoveratric acid** | 7.032368783 | 1.895511593 | 0.010652993 | Up |
| **alpha-ketoglutarate** | 1.214238815 | 1.906715826 | 0.039619943 | Up |
| **Glyceric acid** | 1.615885117 | 2.041744309 | 0.004226619 | Up |
| **D-Mannose** | 2.484017405 | 2.064223392 | 0.000620844 | Up |
| **Ribothymidine** | 2.605238885 | 2.102349346 | 0.029560908 | Up |
| **Adynerin** | 1.707778284 | 2.236952611 | 0.03190423 | Up |
| **Dimethylglycine** | 2.420075051 | 2.343373626 | 0.01242607 | Up |
| **Xanthosine** | 2.73629897 | 2.353605588 | 0.036893521 | Up |
| **D-(+)-Melibiose** | 1.088051004 | 2.36170932 | 0.004364935 | Up |
| **L-Glutamate** | 6.198750145 | 2.403224251 | 0.013760015 | Up |
| **Acamprosate** | 5.126965829 | 2.492220888 | 0.038550317 | Up |
| **Isobutyric acid** | 9.293858727 | 2.795961355 | 0.000524476 | Up |
| **Purine** | 2.82856092 | 2.799980846 | 0.029097886 | Up |
| **D-Ribose** | 1.019861263 | 3.054397448 | 0.016727239 | Up |
| **Citraconic acid** | 1.589485082 | 4.718794883 | 0.031919487 | Up |
| **(S)-2-Hydroxyglutarate** | 2.268473401 | 5.028920703 | 0.02798178 | Up |

**Supplementary Tables 2**|Differential serum metabolites.

| **Name** | **VIP** | **Fold change** | ***P* value** | **Up Down** |
| --- | --- | --- | --- | --- |
| **Xanthosine** | 1.087288166 | 0.374090128 | 0.003418264 | Down |
| **12(R)-HETE** | 13.44242831 | 0.480616487 | 1.57072E-05 | Down |
| **L-Malic acid** | 2.183588976 | 0.508226776 | 5.99825E-05 | Down |
| **D-Allose** | 3.205147405 | 0.545266384 | 0.000266567 | Down |
| **D-Mannose** | 2.77064834 | 0.55824989 | 8.89142E-05 | Down |
| **Myo-inositol** | 1.885525683 | 0.566327762 | 3.90508E-10 | Down |
| **Phosphorylcholine** | 1.99243667 | 0.586674043 | 0.000222641 | Down |
| **Pyruvaldehyde** | 1.308909323 | 0.630527607 | 0.000167148 | Down |
| **Dihydroxyacetone** | 1.410680261 | 0.654635323 | 0.000171794 | Down |
| **D-Lyxose** | 1.149007284 | 0.677261367 | 0.000267522 | Down |
| **Sedoheptulose** | 1.356455879 | 0.690859246 | 0.001276076 | Down |
| **alpha-Linolenic acid** | 6.720910689 | 0.70358617 | 0.002098527 | Down |
| **(4Z,7Z,10Z,13Z,16Z,19Z)-4,7,10,13,1 6,19-Docosahexaenoic acid** | 18.52323102 | 0.720552614 | 0.000140615 | Down |
| **Arachidonic Acid (peroxide free)** | 11.60757311 | 0.744221404 | 0.00213773 | Down |
| **L-Alanine** | 1.264460881 | 0.797148543 | 0.01537648 | Down |
| **DL-lactate** | 6.053627888 | 0.852635368 | 0.039542651 | Down |
| **L-Tryptophan** | 2.443225096 | 1.293362363 | 0.013057223 | Up |
| **2-hydroxy-butanoic acid** | 1.492722503 | 1.459657578 | 0.009446272 | Up |
| **9,10-DiHOME** | 1.446463523 | 1.517244327 | 0.003572403 | Up |
| **3-Phenylpropanoic acid** | 1.475393124 | 1.763222232 | 0.001058652 | Up |
| **Myristoleic acid** | 3.316176563 | 2.159658892 | 0.000148139 | Up |
| **Thymidine** | 4.176316654 | 2.543982328 | 1.7401E-05 | Up |
| **Urocanic acid** | 1.389076597 | 3.176634231 | 1.75077E-08 | Up |
| **2'-Deoxyuridine** | 5.010924732 | 3.249797063 | 5.58017E-06 | Up |
| **Deoxycholic acid** | 1.677527137 | 3.340681925 | 1.47994E-05 | Up |
| **Cholic acid** | 1.585906403 | 5.481984469 | 0.00611367 | Up |
| **Taurochenodeoxycholate** | 9.821796454 | 10.27811042 | 0.004904872 | Up |

1. **Other supplementary materials**

### 3.1 16S rDNA Sequencing

（1）Extraction of genome DNA

Total genome DNA from samples was extracted using CTAB/SDS method. DNA concentration and purity were monitored on 1% agarose gels. According to the concentration, DNA was diluted to 1ng/μl using sterile water.

（2）Amplicon Generation

Primer:The V3-4 hypervariable bacterial 16S rRNA gene region was amplified with the universal primer 338F (5’-ACTCCTACGGGAGGCAGCAG-3’) and 806R (5’-GGACTCANNGGGTA TCTAAT-3’). All PCR reactions were carried out in 30 μl reactions with 15μl of Phusion®High-Fidelity PCR Master Mix (New England Biolabs); 0.2μM of forward and reverse primers, and about 10 ng template DNA. Thermal cycling consisted of initial denaturation at 98℃ for 1 min, followed by 30 cycles of denaturation at 98℃ for 10 s, annealing at 50℃ for 30 s, and elongation at 72℃ for 60 s. Finally, 72℃ for 5 min.

（3）PCR Products quantification and qualification

Mix the same volume of 1X loading buffer (containing SYB green) with PCR products and operate electrophoresis on 2% agarose gel for detection. Samples with the bright main strip between 400-450bp were chosen for further experiments.

（4）PCR Products Mixing and Purification

PCR products were mixed in equidensity ratios. Then, the mixture of PCR products was purified with AxyPrepDNA Gel Extraction Kit (AXYGEN).

（5）Library preparation and sequencing

Following the manufacturer's recommendations, sequencing libraries were generated using NEB Next®Ultra™DNA Library Prep Kit for Illumina (NEB, USA), and index codes were added. The library quality was assessed on the Qubit@ 2.0 Fluorometer (Thermo Scientific) and Agilent Bioanalyzer 2100 system. At last, the library was sequenced on an Illumina Miseq/HiSeq2500 platform, and 250bp/300bp paired-end reads were generated.

**3.2 The analysis and statistics of the metagenomic data:**

The pair-end (PE) double-end sequence data obtained by Miseq sequencing is subjected to quality control processing on the measured Fastq data, and finally, high-quality FASTA data is obtained.

Specific parameters are as follows:

（1）Paired-end reads assemblies

Paired-end reads from the original DNA fragments were merged using FLASH, a fast and accurate analysis tool designed to merge paired-end reads when at least some of the reads overlap the read generated from the opposite end of the same DNA fragment. Paired-end reads were assigned to each sample according to the unique barcodes.

（2）OTU cluster and Species annotation

Sequences analysis was performed by the UPARSE software package using the UPARSE-OTU and UPARSE-OTUref algorithms. In-house Perl scripts were used to analyze alpha (within samples) and beta (among samples) diversity. Sequences with ≥97% similarity were assigned to the same OTUs. We pick representative sequences for each OTU and use the RDP classifier to annotate taxonomic information for each representative sequence. To compute Alpha Diversity, we rarify the OTU table and calculate three metrics: Chao1 estimates the species abundance; Observed Species estimates the number of unique OTUs found in each sample; and the Shannon index. Rarefaction curves were generated based on these three metrics.

（3）Phylogenics distance and community distribution

Graphical representation of the relative abundance of bacterial diversity from phylum to species can be visualized using the Krona chart. Cluster analysis was preceded by principal component analysis (PCA), which was applied to reduce the dimension of the original variables using the QIIME software package. QIIME calculates both weighted and unweighted unifrac distance, which are phylogenetic beta diversity measures. We used unweighted unifrac distance for Principal Coordinate Analysis (PCoA), and Unweighted Pair Group Method with Arithmetic mean (UPGMA) Clustering. PCoA helps to get principal coordinates and visualize them from complex, multidimensional data. It t transforms from a distance matrix to a new set of orthogonal axes. The maximum variation factor is demonstrated by the first principal coordinate, the second maximum one by the second principal coordinate, and so on. UPGMA Clustering is a hierarchical clustering method using average linkage and can be used to interpret the distance matrix.

（4）Statistical analysis

A. QIIME (v1.8.0) was used to generate rarefaction curves and to calculate the richness and

α- diversity indices based on the OTU information, and use R(v3.6.0) software to plot. (R packages alpha.div)

1. Based on the results of taxonomic annotation and relative abundance, R (v3.6.0) software

was used for bar-plot diagram analysis.

1. To describe the dissimilarity between multiple samples, PCA was zanalyzed by R (v3.6.0)

based on the OTU information from each sample. QIIME (v1.8.0) was used for β diversity analysis.The β-Diversity distance matrix between samples were calculated using the Bray Curtis algorithms and plotted PCoA or Unweighted Pair Group Method with Arithmetic Mean (UPGMA) clustering tree. PERMANOVA is a non-parametric variant of multivariate analysis of variance used to compare the differences and similarities of statistical index values of multiple groups of observation samples. It uses the distance matrix to decompose the total variance, analyze the interpretation degree of different grouping factors or different environmental factors to the sample difference, and use the substitution test to carry out the statistical significance analysis of the interpretation of each variable. When the calculated *P* <0.05, the original hypothesis is rejected. That is, the center point or distribution of different groups of samples in the detection space is significantly different.

1. Use Python (v2.7) software for LEfSe analysis.
2. Inter-group difference analysis: Kruskal-Wallis (R (v3.6.0)), metastatic (math (v.1.34.4)), and Wilcoxon (R (v3.6.0)).
3. R packages “heatmap” was used for heatmaps. R packages “cor.test” was used for spearman correlation.

**3.3 The analysis and statistics of the GC-MS data:**

After normalized to total peak intensity, the processed data were analyzed by R package

(ropls), where it was subjected to multivariate data analysis, including Pareto-scaled principal

component analysis (PCA) and orthogonal partial least-squares discriminant analysis (OPLS-DA).

The 7-fold cross-validation and response permutation testing were used to evaluate the robustness of

the model. The variable importance in the projection (VIP) value of each variable in the OPLS-DA

model was calculated to indicate its contribution to the classification. Metabolites with the VIP

value >1 was further applied to Student’s t-test at univariate level to measure the significance of

each metabolite, the p values less than 0.05 were considered as statistically significant.
